# Supplementary material for: Surface Microbiome Profiling of Dental Elevators Using Third‐Generation Sequencing: Implications for Infection Control in Dental Practice
Source: Microbiologyopen. 2025 Dec 8;14(6):e70178. doi: 10.1002/mbo3.70178 (PMC12685768; doi:10.1002/mbo3.70178)
Supplement: Supplementary file 1 — customer_backup. [file MBO3-14-e70178-s001.zip › customer_backup/customer_backup/function_analysis/COG/allsample/K2species/allsample.reabundance_Class2Genus.txt.pdf]

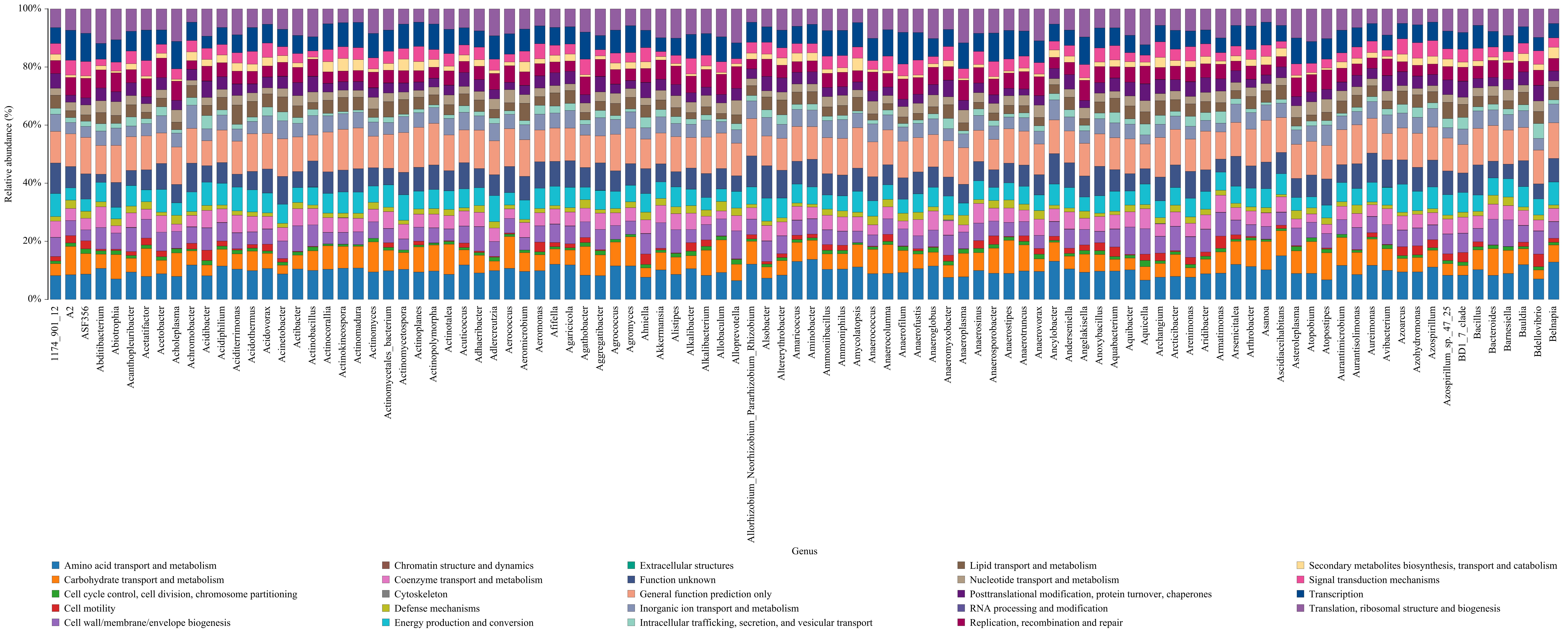

100%
80%
60%
40%
20%
0%
Relative abundance (%)
1174_901_12
A2
ASF356
Abditibacterium
Abiotrophia
Acanthopleuribacter
Acetatifactor
Acetobacter
Acholeplasma
Achromobacter
Acidibacter
Acidiphilium
Aciditerrimonas
Acidothermus
Acidovorax
Acinetobacter
Actibacter
Actinobacillus
Actinocorallia
Actinokineospora
Actinomadura
Actinomyces
Actinomycetales_bacterium
Actinomycetospora
Actinoplanes
Actinopolymorpha
Actinotalea
Acuticoccus
Adhaeribacter
Adlercreutzia
Aerococcus
Aeromicrobium
Aeromonas
Afifella
Agaricicola
Agathobacter
Aggregatibacter
Agrococcus
Agromyces
Ahniella
Akkermansia
Alistipes
Alkalibacter
Alkalibacterium
Allobaculum
Alloprevotella
Allorhizobium_Neorhizobium_Pararhizobium_Rhizobium
Alsobacter
Altererythrobacter
Amaricoccus
Aminobacter
Ammoniibacillus
Ammoniphilus
Amycolatopsis
Anaerococcus
Anaerocolumna
Anaerofilum
Anaerofustis
Anaeroglobus
Anaeromyxobacter
Anaeroplasma
Anaerosinus
Anaerosporobacter
Anaerostipes
Anaerotruncus
Anaerovorax
Ancylobacter
Anderseniella
Angelakisella
Anoxybacillus
Aquabacterium
Aquibacter
Aquicella
Archangium
Arcticibacter
Arenimonas
Aridibacter
Armatimonas
Arsenicitalea
Arthrobacter
Asanoa
Ascidiaceihabitans
Asteroleplasma
Atopobium
Atopostipes
Aurantimicrobium
Aurantisolimonas
Aureimonas
Avibacterium
Azoarcus
Azohydromonas
Azospirillum
Azospirillum_sp._47_25
BD1_7_clade
Bacillus
Bacteroides
Barnesiella
Bauldia
Bdellovibrio
Belnapia
Genus
Amino acid transport and metabolism
Carbohydrate transport and metabolism
Cell cycle control, cell division, chromosome partitioning
Cell motility
Cell wall/membrane/envelope biogenesis
Chromatin structure and dynamics
Coenzyme transport and metabolism
Cytoskeleton
Defense mechanisms
Energy production and conversion
Extracellular structures
Function unknown
General function prediction only
Inorganic ion transport and metabolism
Intracellular trafficking, secretion, and vesicular transport
Lipid transport and metabolism
Nucleotide transport and metabolism
Posttranslational modification, protein turnover, chaperones
RNA processing and modification
Replication, recombination and repair
Secondary metabolites biosynthesis, transport and catabolism
Signal transduction mechanisms
Transcription
Translation, ribosomal structure and biogenesis
